# New “Smart” Systems for Atmospheric Aerosol and Reactive Gas Sampling in Ambient Air

**DOI:** 10.3390/s18113602

**Published:** 2018-10-23

**Authors:** Paolo Cristofanelli, Maurizio Busetto, Enrico Ronchi, Paolo Miatto, Angela Marinoni, Francescopiero Calzolari, Paolo Bonasoni, Luca A. Tagliafico

**Affiliations:** 1 National Research Council of Italy, Institute of Atmospheric Science and Climate, Via Gobetti 101, I-40129 Bologna, Italy; info@mauriziobusetto.com (M.B.); a.marinoni@isac.cnr.it (A.M.); f.calzolari@isac.cnr.it (F.C.); p.bonasoni@isac.cnr.it (P.B.); 2 Maurizio Busetto, Via Martucci, 40126 Bologna, Italy; 3 LEN srl, Via Sant’ Andrea di Rovereto 33 CS, I-16043 Chiavari (GE), Italy; enrico.ronchi@len.it (E.R.); len@len.it (P.M.); 4 DIME, University of Genoa, Via all’Opera Pia, 15, I-16145 Genova (GE), Italy; tgl@ditec.unige.it

**Keywords:** reactive gases, atmospheric aerosol, air sampling, smart technologies

## Abstract

Nowadays a recognized need for accurate observations of atmospheric aerosols (AEs) and reactive gases (RGs) exists in the framework of regional, national and global near-surface networks based on permanent or mobile measurement stations. In this context, a paramount and not-trivial issue is related to the correct execution of continuous sampling of ambient air and its subsequent distribution to measurement analyzers hosted inside the stations. Sampling artifacts must be minimized for obtaining reliable pictures of ambient air composition. To respond to this need, a suite of novel “smart” and relatively low-cost systems for the continuous sampling of ambient air was developed in the framework of the 2012–2015 I-AMICA Project. These systems were designed to execute AE and RG measurements according with WMO/GAW and ACTRIS recommendations and standard operation procedures. A particular attention was dedicated to the stabilization and control of the sampling flow rates and temperatures. The analysis of one full year of operations at the WMO/GAW regional station of Capo Granitola (GAW ID: CGR, Italy), allowed to conclude that these systems are effective in meeting the technical requirements for correct execution of AE and RG measurements.

## 1. Introduction

The provision of reliable observations of the chemical composition and physical properties of the atmosphere is a pillar for understanding atmospheric chemistry and climate change both in term of process investigation and detection of regional and global changes. To assure the reliability of observed data, it is crucial to standardize and increase compatibility of measurements and data: to these aims global (e.g., Global Atmosphere Watch by the World Meteorological Organization—GAW/WMO) and regional (e.g., Aerosol, Clouds, and Trace gases Infrastructure—ACTRIS) efforts were undertaken in the last decades (see [1,2]). The standardization of measurement techniques is an urgent issue especially for atmospheric aerosols (hereinafter AEs) and reactive gases (hereinafter RGs).

AEs influence the energy budget of the atmosphere through direct and indirect radiative effects. Direct effects include the scattering and absorption of radiation in sensible wavelengths, while indirect effects involve the influence of AEs on cloud condensation nuclei (CCN) which in turn affects cloud albedo, lifetime and precipitation frequency. Absorbing AEs (both natural and anthropogenic) also affect climate by deposition on snow and ice surface, thus modifying the albedo of the cryosphere at regional and global scales. Stocker et al. [3] estimated the AEs radiative forcing over 1750–2011 as −0.9 W/m^2^ (90% confidence interval: from −1.9 to −0.1 W/m^2^). This radiative forcing encompasses a dominant cooling effect and a warming contribution mostly from black carbon (a well-known short-lived climate forcer, see [4]).

In addition to climate influence, AEs affect many aspects of human health and the environment. Toxic chemical components within AEs are known to have links to health problems like chronic respiratory and acute cardio-vascular diseases. AEs were also closely linked to visibility reduction, acid rain, and urban smog in many locations of the world. Dimming effect of solar radiation by AEs can induce a decrease of agriculture yields.

As a function of their diameters, two populations of particles, with different sources, sinks, sizes and chemical compositions, generally constitute the tropospheric AEs. The sub-micrometer fraction (“fine particles”) represents the sum of the nucleation (up to about 20 nm diameter), Aitken (20–100 nm) and accumulation modes (0.1–1.0 μm). The coarse fraction, encompasses AE particles with diameter greater than 1.0 μm. The fine fraction of AEs originates from condensation process and from atmospheric gas-to-particle conversion. It is removed by precipitation and dry deposition. On the other hand, the coarse particles are produced by mechanical processes (e.g., soil erosion by wind, dust mobilization by wind and seawater bubble-bursting) and is mainly removed by sedimentation and interaction with the Earth’s surface.

Accurate size-segregated or size-resolved measurements are needed to determine the AEs properties within each of these populations and to investigate their hence effects on climate and air quality. As an instance, the AE particles within the accumulation mode are the most important for direct radiative forcing while nucleation particle observations are useful for investigating particle formation processes. When present in large amount, also coarse AEs strongly affect the scattering of solar radiation. On the other side, the AE particles in the “fine” mode can potentially penetrate deep into the lung, thus playing important roles for human health and diseases.

RGs are a group of atmospheric molecules with short lifetimes (ranging from hours to a few months). Among them, important roles are played by tropospheric ozone (O_3_), carbon monoxide (CO), reactive nitrogen gases (NO_x_), volatile organic compounds (VOC) and reactive sulfur gases (SO_x_). In particular, the RGs regulate the oxidative capacity of the atmosphere as they represent the key source of reactive atomic and free radical species (e.g., O*, OH, HO_2_, RO_2_). In this way, they have indirect impact on the Earth’s atmosphere radiative forcing by affecting lifetime of greenhouse gas. O_3_ is a powerful greenhouse gas [4], while NO_x_, VOC and SO_2_ affect the formation of aerosol particles and clouds [5]. RGs have direct effects on human health and ecosystem integrity; for these reasons they are the object of regulation emission directives in inhabited regions [6]. Reliable long-term observations of RGs are also useful to assess the effectiveness of the adopted air-quality strategies. Indeed, as reported by [5], information of RG spatial distribution and variability is essential for assessing the effectiveness of emission mitigation measures.

The accurate measurement of AEs and RGs represents a challenging task. To correctly determine AE properties it is paramount to minimize artifacts related with the air sampling. At background measurement sites RG mole fractions range from nmol/mol (i.e., ppb) to pmol/mol (i.e., ppt). Furthermore, RGs are often difficult to sample just because of their reactivity. Thus, a pre-requisite for reliable execution of atmospheric AE and RG observations is the adoption of suitable sampling systems able not only to guarantee the correct execution of air sampling (as an instance, at wet or windy environments) but also to minimize artifacts. In particular, the presence of wind gusts or high wind speed can make the air sampling not efficient, while the presence of water vapor condensation inside the inlet can produce interference in the AEs and RG measurements (i.e., hygroscopic growth of aerosol particle and interaction of gas molecules with water droplets).

GAW/WMO and ACTRIS provide standard operation procedures (SOP) and guidelines about the correct design and implementation of sampling systems for AEs and RGs. By the report [7], GAW/WMO recommended that inlets for AEs provide undisturbed and representative aerosol sampling to measurement instrumentation. To this aim, sampled air should be brought into the station laboratory through a vertical stack with an inlet characterized by a sampling efficiency not depending with wind direction or wind speed. Along the stack the sample flow should be laminar to avoid losses of small particles by diffusion and turbulent inertial deposition. A Reynolds number (Re) of about 2000 is indicated as ideal by [7]. Concerning materials, conductive and non-corrosive material must be used (e.g., stainless-steel) in order to not change the size distribution or chemical composition of the aerosol particles in the sampled air. The implementation of humidity control is suggested due to the strong influence of relative humidity (RH) on the size of atmospheric particles: GAW/WMO recommendations are to maintain the RH lower than 40%. For humid “like-tropical” locations as CGR, no agreed standard technique to dry the aerosols exists. In our case, we decided to implement a passive system based on the modest “sensible heating” of sunlight on the external coating of the air-intake without any direct temperature regulation. For AEs, it is also requested to exclude precipitation because of its interference with the measurements: to this aim, the air inlet should be equipped with a cover to avoid the sampling of drizzle and rain.

Concerning RGs, clear indications about air-inlet design have been provided by GAW/WMO (see [8]) and ACTRIS (see [9]) for the execution of NO_x_ and O_3_ measurements. In particular, the inner surface of the inlet line must be smooth, non-porous and inert: it is recommended to use PFA Teflon^©^ or similar material. To avoid vapors condensation, it is recommended to heat the inlet line. The temperature has to be chosen high enough that no condensation occurs but not too high that thermal decomposition of other substances (e.g., peroxide-acetyl-nitrates) become an artefact: controlled heating a few degrees (3–4°) above ambient temperature is suggested. Gas phase processes may also lead to changes in trace gas mixing ratios, because of different conditions in the inlet line compared to ambient. As an instance for NO_x_ observations an issue can be represented by the interaction with O_3_ in the sampling lines (NO titration). Thus, the residence time in the inlet line must be kept as short as possible (recommended is a residence time of less than 5 s, better below 2 s).

In this paper, we will describe two novel sampling systems for the near-surface observations of AEs and RGs that were developed in the framework of the PON/FESr I-AMICA Project (www.i-amica.it). These systems make use of not-standard and low power consumption technical solutions for the management of wind variability (i.e., gusts and high wind speed) together with smart devices for the regulation of sampling flow rates and air temperature, as well as for the recording of diagnostic data. Firstly (Section 2), we provide a technical description of the sampling systems with a particular emphasis on vertical stacks, used material and the adopted solution for the flow and temperature controls. Then (Section 3), we provide evidences of the system performances by analyzing 1 full year of operation at the regional WMO/GAW station at Capo Granitola (Italy), located along the south-west coastline of Sicily. Finally, in Section 4 we summarize and discuss the results.

## 2. Materials and Methods

### 2.1. Design of Vertical Stacks

**Figure 1:**
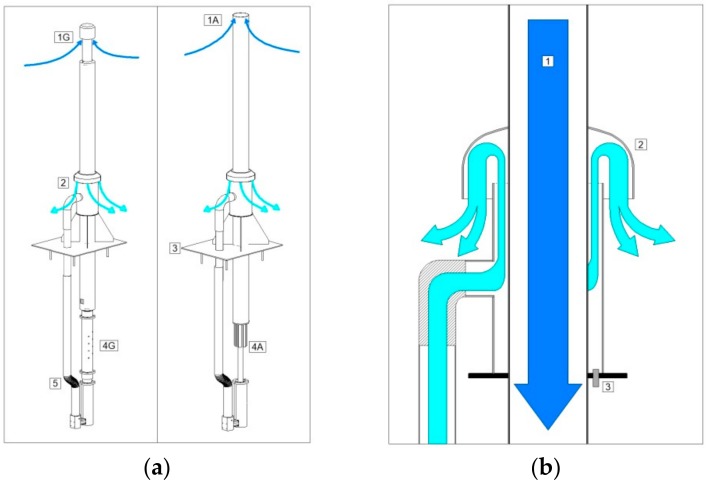
(**a**): air intake systems for RGs (left) and AEs (right). The numbers in the technical diagram denote: air inlet (1), air exhausts (2), flanges for mounting on the roof (3), manifolds for the connection of tubing to analyzers (4), high-velocity fan (5). (**b**): details of the air streams in the proximity of the air exhaust. Dark and light arrows represent the air stream at the inlet and at the exhaust of the intakes, respectively.

Figure 1 shows the air sampling system developed for AEs (a, right) and RGs (b, left). The RG sampling inlet (1G) is equipped with a PFA (Teflon^©^) hat to avoid drizzle and rain sampling, while the AE sampling inlet (1A) is equipped with a size selective (PM_10_) sampling head. The air stream enters the sampling system throughout the inlet points (1G and 1A), flows downward along the vertical stack (ID: 50 mm for the RG system and 100 mm for the AE system) to the manifolds (4G and 4A) where the tubing bringing the sampled air to the analyzers are connected. Then, the air stream passes throughout a high-velocity fan (5, Figure 1a) which sustains the flow rate. Finally the air is thrown outside by the exhaust points (2, Figure 1a). As recommended by WMO/GAW and ACTRIS the vertical stacks is composed by PFA (Teflon^©^, Genova, Italy) for the RG system (externally coated by stainless steel) and by stainless steel AISI 316 for the AE system.

**Figure 2:**
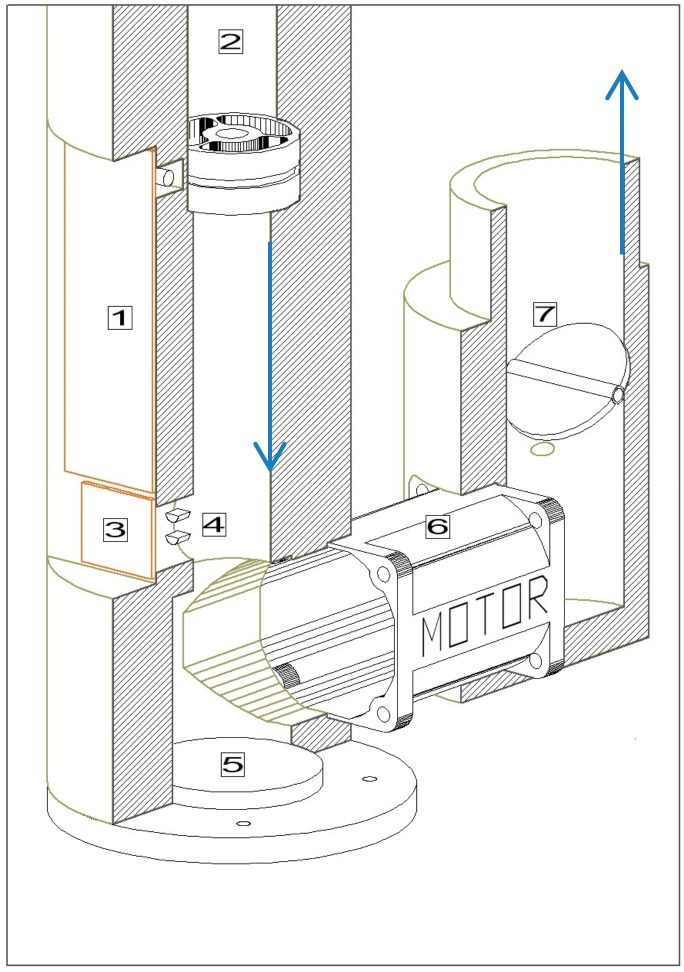
Diagram of the flow generator and control systems: flow control board (1), anemometer (2), T and RH control board (3), T and RH sensor (4), water drainage (5), motor and fan (6), manual air flow shutter (7). The arrows denote the flow direction.

The AE system is equipped with 10 inlets suitable to be connected with 1 tubing with OD of 1” and ½”, 2 tubing with OD of ¾” and 6 tubing with OD with ¼”: to minimize any possible perturbation to the sampling flow, the connection tubing enter the manifold vertically from the bottom of the vertical stack (Figure 2): they extends for 300 mm inside the stack and 200 mm externally to allow the easy connection of conductive silicon tubing. The central pipe represents the connection between the manifold and the system for flow generator and control. A specific tubing is used for the drain of water. The RG system is equipped with 12 inlets suitable to connect tubing (PFA, PTFE, stainless steel, Synflex 2000^©^, Dekabon^©^) with an OD of ¼”. These tubing enter throughout the manifold horizontally and take air perpendicularly in respect to the main air stream (Figure 2).

The air flow is required to be constant for both the sampling systems independently from the external wind variability. To this aim a specific system to counteract possible influence of wind variability on the air sampling efficiency has been implemented (see Figure 1b). The reversal “U” pipe of the air exhaust minimizes the influence of external wind variability because the same dynamic pressure is present at the air inlet (1, Figure 1a) and at the exhaust points (4, Figure 1a). Indeed, in the case of high external wind speed, without the adoption of similar systems, “under pressure” conditions could occur at the sampling inlet that counteracts the sucking action of the flow generator and thus can lead to a flow rate decrease or to an over-duty of the flow generator (in the case the sampling flow is controlled). The shield above the “reversal U” pipe together with a long-enough distance between the inlet and exhaust points, ensure that the air passing through the system will not be sampled again.

### 2.2. Flow Generation and Control

The system developed for the flow generation and control is reported in Figure 2: the air stream enters from the anemometer (2) and it is discharged from the manual shutter (7). The anemometer is used to measure the air velocity inside the pipe connecting the manifold to the flow generator and control system. The air velocity value is continuously compared with the set-point value needed to maintain the constant flow in the air intake (see Section 3). If the measured air velocity is lower (greater) then the set-point value, the motor (6, Supermicro Axial Fan 40 mm by Nidec^©^, Kyoto, Japan) decreases (increases) the fan rotation frequency until target values is reached. The manual air shutter (7) was implemented to compensate possible impedance disembalance due to the different geometry of the air inlet and exhaust points. The opening diameter of the shutter, especially for the AE system that can be equipped with various size selective sampling heads, has to be regulated only at the beginning of the system operation.

### 2.3. Sampling Temperature Control

**Figure 3:**
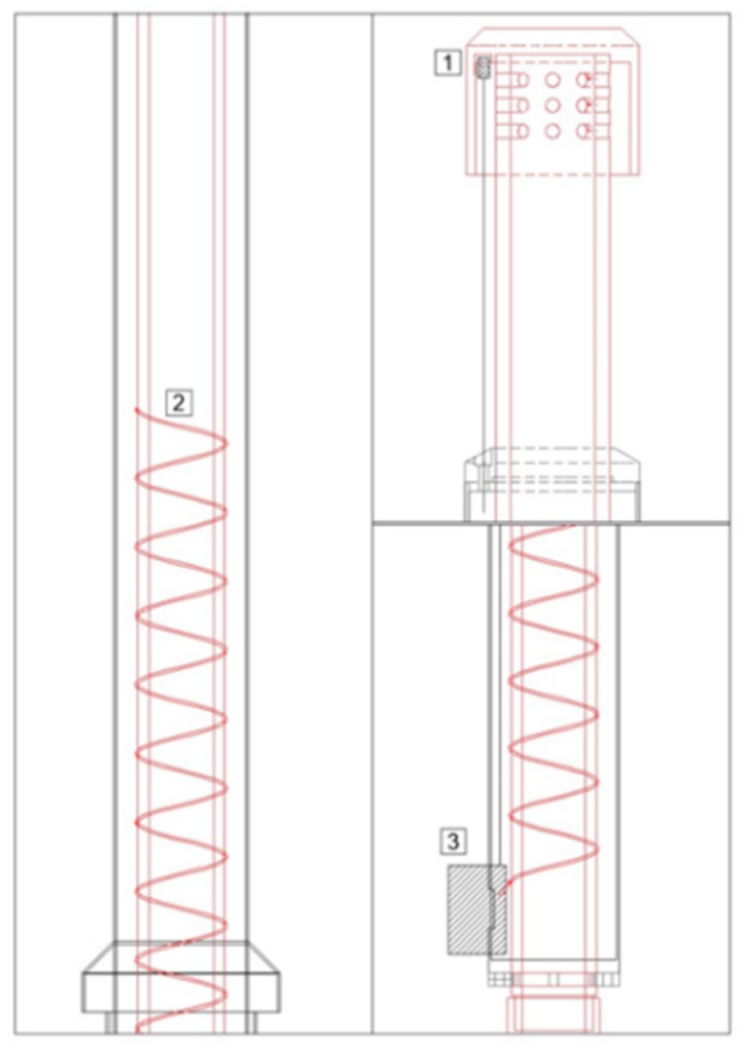
Diagram of the RG temperature control system: external temperature sensor (1), heating system (2), electronic controller board (3).

**Figure 4:**
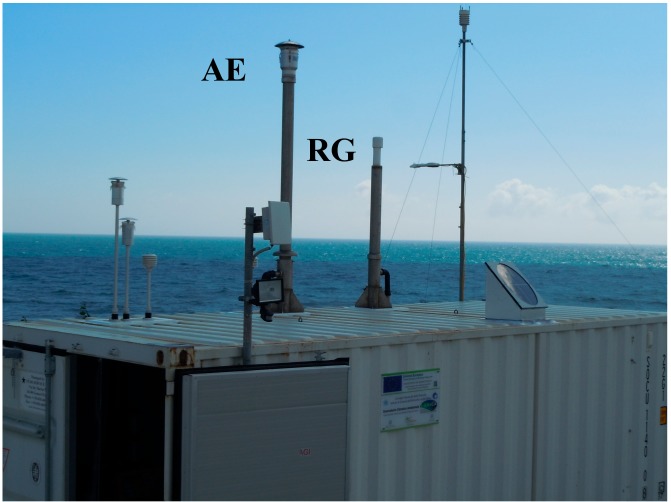
External view of the WMO/GAW regional station at Capo Granitola (CGR): AE and RG sampling systems are indicated.

For the RG system an important constraint is related to the need to avoid water condensation in the air intake by maintaining the internal temperature 3–4 °C higher than the external air-temperature. Figure 3 provides a description of the system used to control the temperature of the air intake. A heating resistor (2, Figure 4) is implemented inside the wall of the RG air intake. The resistor is activated by a control board (3, Figure 4) when the difference between internal and external temperature is less the 2 °C and deactivated when it is more than 4 °C. The air-temperature within the air-intake (*T_gas_*) is continuously measured by a thermometer placed just below the manifold, while the external air-temperature (*T_ext_*) is measured by a thermometer close to the inlet point (1, Figure 4).

### 2.4. Data Acquisition System and Control Boards

The control and data acquisition systems are based on the use of “smart” and low-cost miniaturized computers. In particular, two Raspberry PI3 model B are used to continuously monitor the performance of the RG and AE sampling systems as well as to allow their remote control (e.g., regulation of fan rotation velocity). Raspberry PI is a family of single-board computers widely used in educational and smart applications (see https://www.raspberrypi.org/). These “smart” computers are equipped with four USB ports, a Quad Core CPU, 1 GB Ram and wireless LAN connection. Thanks to their very low weight (about 140 g), small dimensions (12.2 × 7.6 × 3.4 cm), very low power consumption (1.2 W) and affordable cost (about 40.00 euros), Raspberry^©^ devices are very suitable for use in remote and unattended situations. Our systems are equipped with the *Raspbian* operating system, while *WiringPi* library (released under the GNU Lesser General Public License, see http://wiringpi.com/) is used to connect the computers with sensors. During the period of test, no major failures of these control and data acquisition systems were recorded: this further stresses the suitability of usage of these “smart” devices even for continuous “high-demanding” monitoring activities.

## 3. Results: 1 Year of Field Operations

### 3.1. The Measurement Site

Capo Granitola (37.66670° N 12.65000° E; 5 m a.s.l.) is located at the southern Sicily coastline facing the Strait of Sicily, at Torretta Granitola (12 km from Mazara del Vallo, 52,000 inhabitants), within the scientific campus of the Institute for the Marine-Coastal Environment (CNR-IAMC). A WMO/GAW regional station (ID: CGR) was established in 2015 in the framework of the PON/FESR Project I-AMICA with the purpose of carrying out continuous atmospheric composition measurements representative of western Sicily/central Mediterranean basin conditions and providing data to investigate the influence of specific atmospheric processes (e.g., long-range air mass transport, mineral dust emitted from Northern Africa, anthropogenic ship emissions) on the variability of trace gases [10] and aerosol in the Mediterranean region. CGR observatory is located on a cliff extending about 10 m from the sea surface (Figure 4).

**Figure 5:**
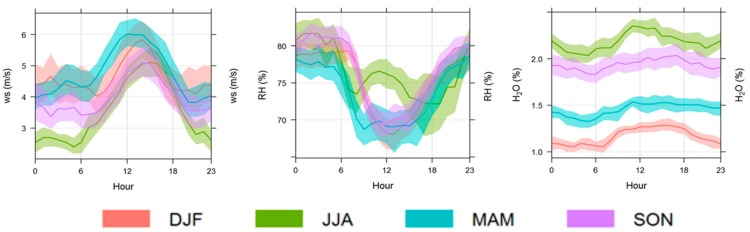
Seasonal average diurnal variation of wind speed (WS), relative humidity (RH) and water vapor (H_2_O) at CGR during one full year. Dashed areas denote the 96% confidence levels. Time is expressed in local time (UTC + 1) (adapted by [10]).

Thus, it is directly exposed to resuspension of sea water droplets, sea salt dust particles from the seaside. This location is affected by the sea-land breeze regime, with prevailing (49% of hourly occurrence throughout the measurement period) gentle wind breezes (up to 4 m/s) from inland (NW-NE) during the night and prevailing (80%) winds from the sea (W-SE) during daytime. The systematic occurrence of the breeze regime is also well-reproduced by the seasonal diurnal cycle of wind speed (Figure 5). On a seasonal basis, high water vapor values (on average higher than 2%) were observed during summer (Figure 5), when a clear diurnal cycle with the highest value during daytime is also observed at the station. Events of high wind speed (i.e., hourly mean averaged values higher than 10 m/s) associated with the presence of synoptic-scale disturbances are observed at this measurement site for about 4% of observations. These environmental conditions (high presence of local re-suspended/emitted aerosol, high water vapor ambient mixing ratio, occurrence of high wind speed) make this site a challenging location to test the inlet systems for atmospheric constituents continuous sampling. More details about the measurement site can be found at the web site http://www.i-amica.it/i-amica/?page_id=632&lang=en, while measured data can be found at https://geonetwork.igg.cnr.it.

### 3.2. System Performance Analysis

#### 3.2.1. Sampling Flow Control

In order to keep the residence time of the gas samples in the air intake below 2 s, according to Equation (1), the sampling flow rate must be higher than 240 L min^−1^. In Equation (1), V_inlet_ denotes the total volume of the air intake (7.9 L), t_res_ is the residence time (<2 s) and Φ_gas_ represents the sample flow rate (L min^−1^):

(1) $$\Phi_{\mathrm{gas}}=V_{\mathrm{inlet}}/t_{\mathrm{res}}$$

As requested by GAW/WMO guidelines, for the AE sampling the air flow must be laminar (i.e., Reynolds number Re ranging from 1000 to 2000) in the aerosol intake. For a pipe with diameter d, Re is defined as:

Re = ρdv/μ(2)

where ρ s the air density (1.185 kg m^−3^), μ the air dynamic viscosity (1.831 × 10^−5^ kg m^−1^ s^−1^) and v the mean air velocity. From Equation (2), we can calculate the flow rate Φ_AE_ as function of Re:

Φ_AE_ = μπd/4ρ Re (3)

According to Equation (3), the air flow rate in the aerosol intake must be maintained between 73 L min^−1^ (Re = 1000) and 145 L min^−1^ (Re = 2000) to guarantee a laminar regime.

The pneumatic component of AE and RG sampling systems was designed to maintain the sampling flow rate and temperature as constant as possible, even in presence of significant wind and ambient temperature variability occurring over different time scales (i.e., from minute to seasons).

**Figure 6:**
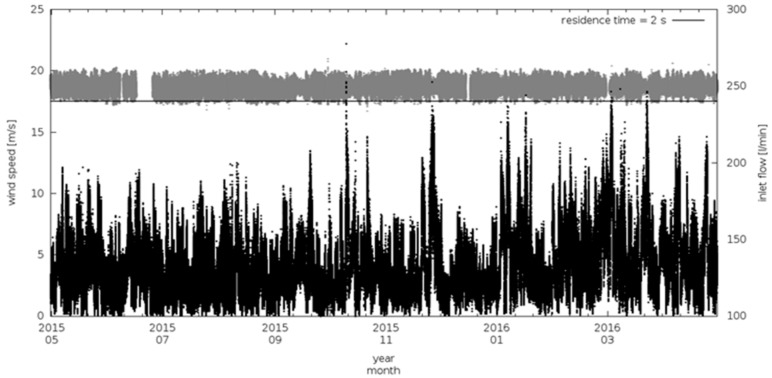
Time series of sampling flow of trace gas intake (grey) and wind speed (back) at CGR (1-min averages). The top black straight line represents the minimum allowed flux to maintain the air residence time below 2 s in the air intake.

**Figure 7:**
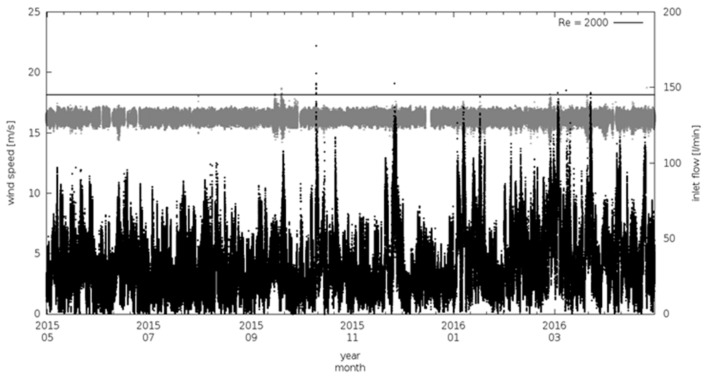
Time series of sampling flow of the aerosol intake (grey) and wind speed (back) at CGR (1-min averages). The top black straight line represents the maximum allowed flux to maintain a laminar regime in the aerosol air intake.

Figure 6 and Figure 7 report one full year (May 2015–May 2016) of 1-min average values of gas and aerosol inlet flows together with wind speed measured at CGR by a WXT520 meteorological station (Vaisala, Finland).

During the considered period, less than 10% of flow data are missed, mostly due to power interruption at the station and inlet or blower maintenances. The sample flow rates, both in the gas and in the aerosol air-intake, have been characterized by a constant mean values during the time, with average value well centered around the target flow rates.

**Figure 8:**
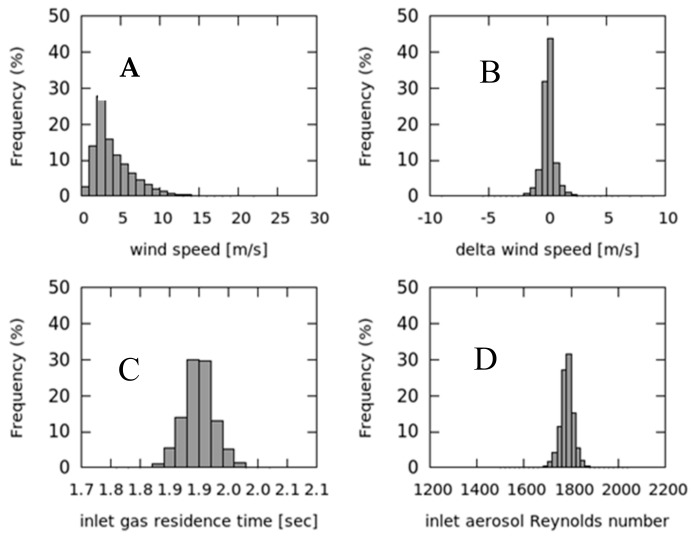
Frequency distributions of wind speed (**A**), wind speed variability (**B**), sample residence time calculated for the gas air-intake (**C**) and Re calculated for the aerosol air-intake (**D**).

To evaluate the possible impact of wind gusts on flow rate stability, we calculated the “wind variability” (δ_ws_), i.e., the difference between two consecutive wind speed 1-min values. Figure 8 reports the frequency distribution of t_res_ and Re over the investigation period, calculated respectively from Equation (1) and (3) using the measured air velocity, together with frequency distribution of wind speed and δ_ws_. During the considered period, wind speed ranged from 0 up to 15 m s^−1^ with a rather narrow δ_ws_ distribution, thus indicating that CGR is only slightly affected by the occurrence of wind gusts but that significant wind speed variability occurred on synoptic (day to day) or local (diurnal) scales. Both t_res_ and Re showed a very narrow statistical distribution (see also Table 1) centered at 1.9 s and 1782, respectively. This suggests that the system design is effective in maintaining the actual flow conditions within the prescribed target values. t_res_ and Re standard deviations are less than 2% of their respective mean value, indicating a very good stability of the flow rate and a small (random) variability: only 1% of the data-set for t_res_ and Re did not meet the target values.

**Figure 9:**
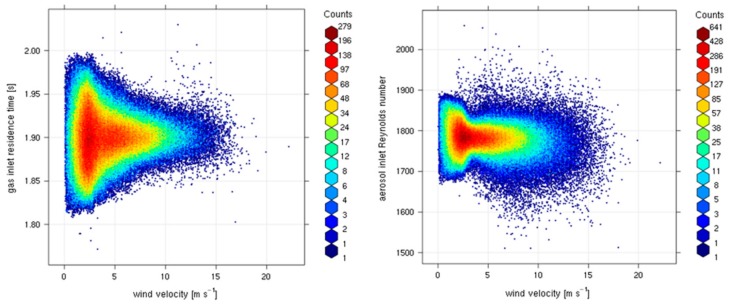
Bivariate analysis of t**_res_** (left plot, *y*-axis) and Re (right plot, *y*-axis) as a function of wind speed (*x*-axis). The colored scale (see legends) represents the number of occurrences.

To better investigate the dependence of flow rate with external wind speed variability, we analyzed t_res_ and Re as a function of wind speed. Figure 9 does not show any evident relationship between t_res_ and Re and external wind speed. Nevertheless, the t_res_ and Re data dispersion decrease for increasing wind speed values. Even if it can be argued that this can be a statistical artifact related with the relatively low number of observations for increasing values of wind speed, this is a further hint supporting the ability of the proposed system in maintaining stable sampling flows under different wind regimes.

#### 3.2.2. Sampling Temperature Control (RG)

**Figure 10:**
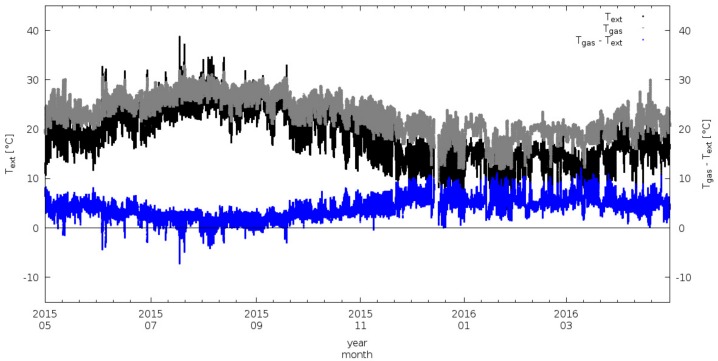
Time series of ambient air temperature (*T_ext_,* black), internal temperature of gas intake (*T_gas_*, grey) and their differences (*T_gas_*-*T_ext_*, blue). 1-min average values are reported.

As specified in Section 2, the RGs air-intake is equipped with a heating system able to actively regulate the internal inlet temperature to avoid condensation inside the air-intake and volatilization of aerosol chemicals. The system must be able to keep the inlet temperature about 4 °C higher than ambient temperature (*T_ext_*). Figure 10 reports one full year (May 2015–May 2016) of 1-min average values of internal temperature of the trace gas air intake (*T_gas_*) and *T_ext_*. From the inspection of these time series, it is evident that the system was effective in maintaining the trace gas inlet temperature higher than the external air temperature.

**Figure 11:**
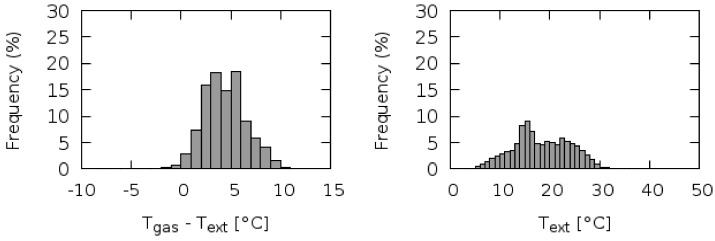
Frequency distributions of difference between internal gas intake temperature and external ambient temperature (**left**) and external air temperature (**right**).

**Figure 12:**
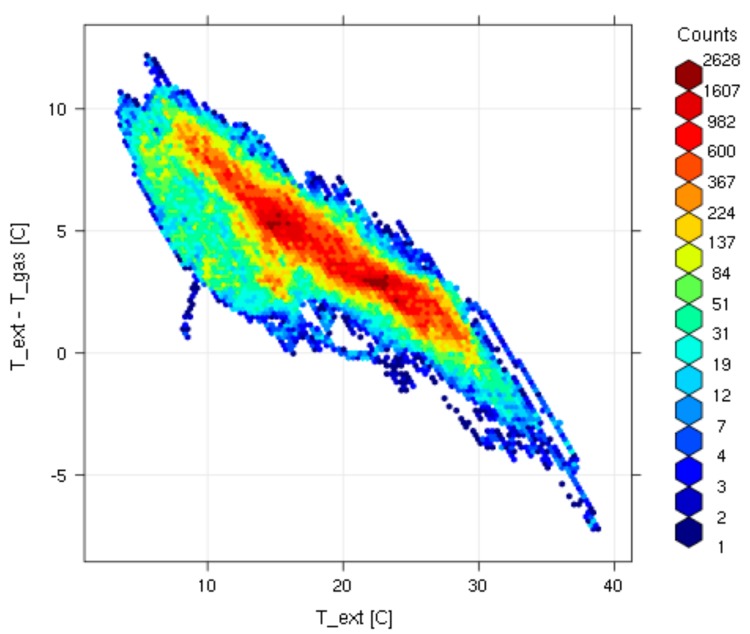
Bivariate analysis of *T_ext_*-*T_gas_* (*y*-axis) as a function of *T_ext_* (*x*-axis). The colored scale (see legends) represents the number of data occurrence.

The statistical analysis of the temperature difference (*T_gas_*-*T_ext_*) is characterized by a Gaussian distribution centered at 4.3 °C, i.e., around the target values requested by ACTRIS (Figure 11). Only for 1.4% of the analyzed data-set, the system fails in maintaining the inlet gas temperature higher than the external air temperature: these few cases occurred during hot summer conditions at Capo Granitola, when ambient temperature exceeded 30 °C. This is clearly showed by the cross correlation analysis of temperature values (gas intake vs ambient air, Figure 12): the higher the ambient air temperature, more difficult for the system was to keep a temperature gradient of 4 °C. However, it should be keep in mind that for avoiding volatilization processes of nitrates from the aerosol to the gas phase and thermal decomposition of other trace gases, excessive heating of the air intake must be avoided.

## 4. Summary and Discussion

In the framework of the I-AMICA Project (www.i-amica.eu), we developed novel “smart” and relatively low-cost systems for the continuous sampling of ambient air devoted to the investigation of AE and RG variability, according with guidelines of leading internal programme for the investigation of air-composition variability (i.e., WMO/GAW and ACTRIS). These systems were designed to feed multiple instrumentation for the measurements of RG mixing ratios and AE physical properties (number size distribution, absorption and scattering coefficient, total particle number concentration). A particular attention was dedicated to the implementation of systems for the generation and control of the sampling flow rates and temperatures. In respect to “traditional” rotative or diaphragm pumps and turbo blowers, “low-cost” fans (typically used for computer cooling) are used to generate the sampling flow. These sampling fans can be easily accessed to perform preventive check or substitution in the case of damages. During the first period of system operation at CGR, a few sampling interruptions were experienced due to the damage of the wire welding of the fan motors. This problem was solved by apply an appropriate resin coverage to the electric contacts which prevent any oxidation due to the exposure of high level of sea-salt.

Moreover, in order to minimize “over-pressure” or “under-pressure” conditions, an innovative pneumatic design was applied to the air-intake (see Section 2.1). As testified by one full year of operation at the WMO/GAW “CGR” coastal station, the proposed systems were effective in maintaining sampling flow rates and temperature to defined set-point values independently by external wind speed and external ambient temperature.

“Smart” and low-cost miniaturized computers (i.e., Raspberry PI) are used as control boards to monitor and record the system internal parameters (i.e., flow rates, sample temperature and relative humidity, power consumption, fan motor r.p.m.) as well as external conditions (wind speed, ambient temperature) which make easy the near-real time upload of recorded data as well as the remote control of the system (e.g., regulation of fan rotation velocity). Together with the very low electrical consumption (under 10 W), this makes the proposed systems very suitable for remote sites where the electrical and man powers are limited. Moreover, the following benefits can be obtained by using these smart technologies:

- Low implementation costs
- Easily obtain spare-parts or prepare “back-up” systems
- Possibility to interact (as in the case of Raspberry^©^ computers) with a wide user community for problem solving
- Feasible maintenance even by not-experts
- Possibility of remote diagnosis and setting-up

Further implementations are on-going to adapt these systems to polar or high-mountain environments. The density and viscosity variation with pressure, temperature and absolute humidity is of the order of 10%, and therefore negligible in Re calculations from the point of view of flow control needs. However, we will consider the possibility to include pressure and temperature readings in the calculation of the flow rate needed to maintain “laminar” conditions inside the AE vertical stack.

## Figures and Tables

**Table 1:** Statistical parameters of wind speed (WS), wind variability (δ_ws_), sample residence time (t_res_) calculated for the gas air-intake and Reynolds number (Re) calculated for the aerosol air-intake over the investigation period.

Parameter	Mean	Median	Min	Max	5th Percentile	95th Percentile	Standard Deviation	Number of Data
WS [m/s]	4.0	3.2	0.0	22.2	1.2	9.0	2.5	523,004
δ_ws_ [m/s]	0	−0.2	−8.7	9.3	0	0.9	0.5	523,004
t_res_ [s]	1.90	1.90	1.77	2.03	1.86	1.94	0.03	490,543
Re	1782	1783	1513	2058	1734	1829	29	488,168
